# Assessment of the success and survival of full mouth rehabilitations: a 3 year follow up study

**DOI:** 10.1016/j.jobcr.2025.10.007

**Published:** 2025-10-15

**Authors:** Divyansh Sinha, Suresh Venugopalan, Vijay Anand

**Affiliations:** Department of Prosthodontics and Implantology, Saveetha Dental College and Hospitals, Saveetha Institute of Medical and Technical Sciences (SIMATS), Saveetha University, Chennai, 600077, Tamil Nadu, India

**Keywords:** Full mouth rehabilitation, mUSPHS, Occlusion, Occlusal plane, Aesthetic

## Abstract

**Introduction:**

Full mouth rehabilitation (FMR) addresses complex dental issues like tooth wear, cosmetic concerns, and loss of occlusal stability, enhancing both function and aesthetics. This study aimed to evaluate the success and survival of tooth-supported FMR treatments and identify common causes of treatment failure.

**Methodology:**

A retrospective analysis of 60 patients who underwent FMR between 2019 and 2021 was conducted. Data were collected from the Dental Information Archiving Software (DIAS) and analyzed using modified USPHS criteria, covering parameters like postoperative sensitivity, anatomical form, and marginal adaptation, marginal discoloration, surface texture and recurrent caries. Additional aesthetic and functional discrepancies were also documented.

**Results:**

At cementation, all parameters scored 100 % alpha. At 3-year follow-up, slight declines were observed in sensitivity (99.17 % alpha), anatomic form (99.17 % alpha), marginal discoloration (98.88 % alpha), and recurrent caries (99.17 % alpha), with no statistical significance. Marginal adaptation (97.22 % alpha, *p* = 0.004) and surface texture (97.5 % alpha, *p* = 0.006) showed significant differences. Aesthetic issues included midline disparities (34 %) and ceramic chipping (30 %), while functional issues included absence of incisal guidance (23 %) and occlusal plane discrepancies (16 %).

**Discussion:**

Findings suggest that successful FMR relies on a multidisciplinary approach and advanced CAD/CAM technology, which enhances accuracy and reduces treatment time. However, common aesthetic issues, such as gingival zenith variations, highlight areas for further improvement.

## 1 Introduction

Prosthodontics covers the treatment and restoration of anomalies, enhancing both speech and mastication while providing the patient with an aesthetically pleasing look. One of the most remarkable dental treatments for complications linked to tooth surface loss, cosmetic adjustments, endodontic lesions, periodontal complications, loss in vertical dimension, and issues related to the temporomandibular joint is full mouth rehabilitation.^1^, ^2^, ^3^ Tooth wear, which can have either physiological or pathological causes, is a condition that is defined by tooth surface loss (TSL) in the absence of microbial infection. Although physiological tooth wear happens progressively with age, pathological wear is typically complex in character and could be tough to pinpoint to an exclusive etiological component clinically.^4^ 'Tooth surface loss', coined by Eccles in 1982 was used to incorporate every form of wear disregarding their identity.^5^ Excessive tooth wear is capable of resulting in psychological, cosmetic, and functional difficulties, including reduced vertical dimension of occlusion (VDO).^6^ Treatment of attrited teeth can vary from preventative and targeted therapies to full mouth rehabilitations (FMR) depending on the extent of damage. Stable occlusion is the most vital element in determining the effectiveness of full mouth rehabilitation. It is reliant on the established occlusal concepts and functional harmony between the components of the hard and soft tissues. After recognizing the etiological factor for tooth wear, a diagnostic analysis involving the vertical dimension at occlusion and communication with the patient to identify the necessity of the rehabilitation, with a stepwise planned treatment protocol.^2^ The goal demands a categorization to establish the condition of the current ailment so as to develop a definitive therapy. The classification of occlusal wear is important when communicating with a clinician and to implement a specific approach for treatment. Turner and Misserlian developed a classification in 1984, which is being applied the most commonly. It categorizes patients into 3 categories, namely, class 1 with excessive loss of VDO, class 2 with excessive wear of teeth without loss of VDO but with space available, and class 3 with excessive wear of teeth without loss of VDO but with no space available.^7^ The doctor needs to choose the occlusal approach and then pick an appropriate occlusal concept after examining and classifying the patient's existing clinical state but before initiating the rehabilitative treatment.^3^

FMR serves as an immense difficulty for dental clinicians, necessitating a consistent and reproducible workflow. Incorporating computer aided design/computer assisted manufacture (CAD/CAM) technology, modern prosthodontic dental materials, adhesive dentistry provides an efficient approach towards FMR.^8^^,^^9^ Traditional approaches are frequently lengthy and tedious, involving several clinical visits, with the danger of mistakes in every stage that may necessitate repetition of steps in the treatment. Technological developments in extraoral and intraoral scanning, in addition to smart software development, have considerably made the CAD/CAM production processes more rapid and precise.^10^ This technological application of CAD/CAM increases the accuracy and efficacy of FMR while lowering the duration of treatment and clinical chair-side time.^11^ Determining the long-term success of full mouth rehabilitation involves assessing various factors related to both the functional and aesthetic outcomes of the treatment. The sustained effectiveness of full mouth rehabilitation is determined by evaluating several parameters connected to the treatment's functional and cosmetic outcomes. They involve restoration integrity, health of abutment teeth, TMJ health, occlusion, dental health care, quality of life, awareness for patients, and outlook on therapy.^12^

While numerous case reports, case series, and narrative reviews have addressed various occlusal concepts, materials, and technologies associated with full mouth rehabilitation, the majority focus either on implant-supported prostheses or theoretical frameworks.^1^^,^^12^ To date, few studies have systematically evaluated the long-term clinical outcomes of tooth-supported full mouth rehabilitations using standardized criteria. Moreover, retrospective analyses assessing both structural integrity and patient-centered functional or aesthetic complications remain scarce. Therefore, this study aims to evaluate the 3-year clinical performance, survival, aesthetic and functional causes of failure in completely tooth-supported full mouth rehabilitations using the modified USPHS criteria. The null hypothesis was that there will be no statistically significant difference in the clinical success and survival rates, along with aesthetic and functional changes of tooth-supported full mouth rehabilitations over a 3-year period, as evaluated using the modified United States Public Health Service (mUSPHS) criteria.

### 1.1 Materials and Methods

A retrospective cohort study was conducted at the institutional level to evaluate the clinical outcomes of tooth-supported full mouth rehabilitations (FMRs). The study employed a retrospective consecutive sampling method, where patient records for all those who underwent tooth-supported full mouth rehabilitation at the institution were retrieved from the institutional Dental Information Archiving Software (DIAS) database, covering the period from January 1, 2019, to June 30, 2021. Patients aged between 25 and 75 years who had undergone full mouth rehabilitation procedures with a minimum follow up period of 36 months were chosen and reached out to. Patients with implant restorations and removable prostheses were excluded. Out of the 78 eligible patients, 8 had relocated, 7 could not be contacted after three attempts, and 3 declined participations, resulting in a final sample size of 60 patients, which conformed to the sample size calculation using GPower 3.0 software with power of the study at 0.9. The level of significance was kept at 0.05. Informed permission was acquired in writing from each patient.

Each patient was recalled for clinical evaluation in a standardized manner. Representative teeth were selected from each sextant for analysis: First molars (or nearest functional abutment if missing or replaced by a pontic) were chosen as the representative teeth for sextants 1, 3, 4 and 6 whereas the right central incisor (or adjacent abutment if replaced by a pontic) was chosen for sextants 2 and 5. Assessment was carried out using the modified US Public Health Service (mUSPHS) criteria as presented in Table 1. The mUSPHS criteria include postoperative sensitivity, anatomical form, marginal adaptation, marginal discolouration, surface texture, and recurrent caries. The classifications are categorized as Alpha, Bravo, and Charlie. Additionally, distinct aesthetic inconsistencies, including chipping or fracturing of veneering porcelain, midline deviations, visibility and gingival zenith discrepancies, as well as occlusal irregularities such as incisal guidance, overjet, overbite, key of occlusion, and occlusal plane, were individually recognized in patients and documented. The criteria were explained and illustrated using a standardized training module consisting of photographic slides and definitions, to ensure inter-rater reliability. Two prosthodontists independently evaluated all patients. Prior to data collection, both examiners were calibrated using a training set of 20 representative clinical images covering each USPHS criterion and grading level. Discrepancies between examiners were resolved through a consensus discussion held immediately following each clinical session. If consensus could not be reached, a third senior prosthodontist served as the adjudicator.

**Table 1:** Modified USPHS criteria.

Category	Rating	Criterion
**Postoperative sensitivity**	Alpha (A)	None
	Charlie (C)	Present
**Marginal adaptation**	Alpha (A)	Closely adapted, no visible crevice
	Bravo (B)	Visible crevice, explorer will penetrate
	Charlie (C)	Crevice in which dentin is exposed
**Anatomic form**	Alpha (A)	General contour of the restorations follow the contour of the tooth
	Bravo (B)	General contour of the restorations does not follow the contour of the tooth
	Charlie (C)	The restoration has an overhang
**Marginal discolouration**	Alpha (A)	No discolouration
	Bravo (B)	Superficial staining less than half of the circumferential margin
	Charlie (C)	Deep staining more than half of the circumferential margin
**Surface texture**	Alpha (A)	No surface defects, similar to surrounding enamel
	Bravo (B)	Minimal surface defects, rougher than surrounding enamel
	Charlie (C)	Severe surface defects
**Recurrent caries**	Alpha (A)	No caries present
	Charlie (C)	Caries present

### 1.2 Statistical analysis

Data were subjected to statistics using SPSS 23.0 software. According to the Kolmogrov-Smirnov test of normality, data was found to be not normally distributed. Preoperative and postoperative scores for each parameter were compared using the nonparametric Wilcoxon Signed Ranks test. Interexaminer reliability was confirmed by a Cohen's Kappa statistic of 0.90, indicating high inter-examiner agreement.

## 2 Results

**Fig. 1:**
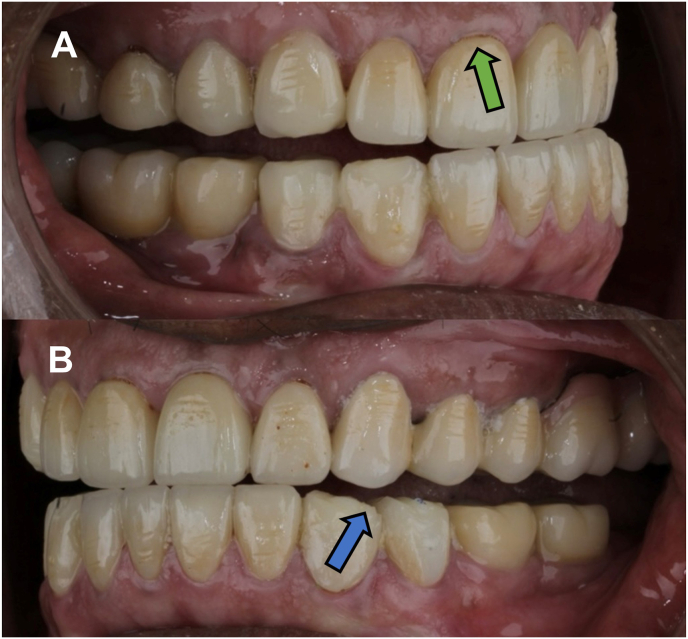
Three year follow up appointment for observation of changes.

The changes observed in the tooth supported FMRs after 3 years of follow up are shown in Fig. 1, with marginal discrepancy seen in maxillary right central incisor, denoted by the green arrow. Generalised ceramic chipping and flattening of occlusal tables can also be observed, denoted by the blue arrow. As shown in Table 2, scores at the time of cementation were 100 % alpha for all the six parameters. At 3 years follow up, postoperative sensitivity dropped to 99.17 % alpha and 0.83 % charlie scores (Z = −1.732, p = 0.083). Anatomic form dropped to 99.17 % alpha, 0.27 % bravo and 0.55 % charlie scores (Z = −1.633, p = 0.102). Marginal discolouration dropped to 98.88 % alpha, 0.27 % bravo and 0.83 % charlie scores (Z = −1.890, p = 0.059). Recurrent caries dropped to 99.17 % alpha and 0.83 % charlie scores (Z = −1.732, p = 0.083). The above parameters showed no statistical difference. However, data was statistically significantly different for marginal adaptation and surface texture. At follow-up, marginal adaptation had 97.22 % alpha, 1.11 % bravo and 1.66 % charlie scores (Z = −2.889, p = 0.004) and surface texture had 97.5 % alpha, 1.38 % bravo and 1.11 % charlie scores (Z = −2.739, p = 0.006) as shown in Table 3.

**Table 2:** Frequency distribution of observed data.

Parameters	Grading	Frequency Baseline	Frequency 3 years
**Postoperative sensitivity**	Alpha	360	357
	Charlie	0	3
**Anatomic form**	Alpha	360	357
	Bravo	0	1
	Charlie	0	2
**Marginal adaptation**	Alpha	360	350
	Bravo	0	4
	Charlie	0	6
**Marginal discolouration**	Alpha	360	356
	Bravo	0	1
	Charlie	0	3
**Surface texture**	Alpha	360	351
	Bravo	0	5
	Charlie	0	4
**Recurrent caries**	Alpha	360	357
	Charlie	0	3

**Table 3:** Wilcoxon signed rank test.

	POST sensitivity - PRE	POST anatomy - PRE	POST marginadapt - PRE	POST margincolour - PRE	POST surface - PRE	POST caries - PRE
**Z**	−1.732^b^	−1.633^b^	−2.889^b^	−1.890^b^	−2.739^b^	−1.732^b^
**Asymp. Sig. (2-tailed)**	0.083	0.102	.004*	0.059	.006*	0.083

^a^. Wilcoxon Signed Ranks Test.

^b^. Based on positive ranks.

**Fig. 2:**
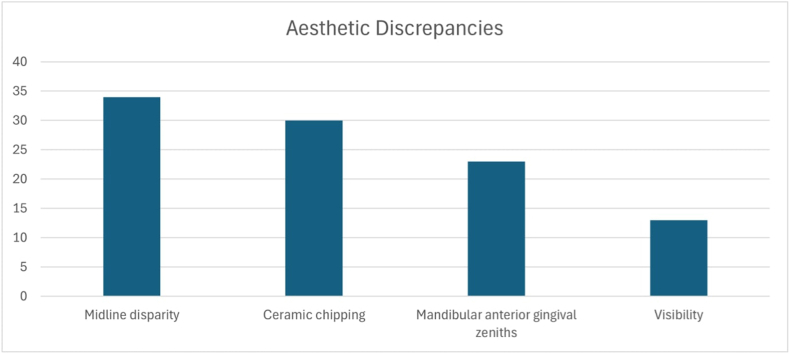
Prevalence of aesthetic discrepancies.

**Fig. 3:**
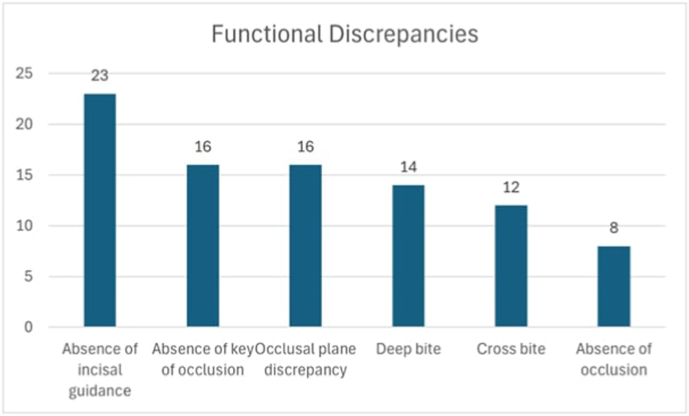
Prevalence of functional discrepancies.

Additional parameters were categorized into aesthetic and functional, and their prevalence was reported as shown in Fig. 2, Fig. 3. The most common aesthetic discrepancy was seen to be midline disparities at 34 %, followed by ceramic chipping 30 %, lower gingival zeniths 23 % and visibility at 13 %. Functional parameters were reported to be absence of incisal guidance 23 %, absence of key of occlusion 16 %, occlusal plane discrepancies 16 %, deep bite 14 %, cross bite 12 % and absence of occlusion 8 %.

## 3 Discussion

A prosthodontist's job of designing and carrying out the corrective rehabilitation of a collapsed occlusion is perhaps one of the most theoretically and functionally hard ones. To be able to detect the requirement for the restoration and construct a detailed treatment plan, the diagnostic information, which includes the occlusal vertical dimension, is examined.^8^ Multidisciplinary approach offers a thorough examination and individualized treatment planning adapted to the patient's requirements.^8^^,^^13^ The widespread tooth wear in a person may be addressed efficiently by a complete mouth rehabilitation strategy. It is of uttermost necessity to assess the existent difference in vertical dimension at rest and occlusion to calculate the appropriate postoperative end point of the prosthesis by keeping the freeway space.^14^

The modified USPHS scale for assessing dental prostheses, including crowns, adopts a simpler grading system of Alpha, Bravo, Charlie whereby Alpha refers to an ideal clinical situation, Bravo refers to clinically acceptable, and Charlie refers to clinically unacceptable.^15^ Postoperative sensitivity, recurrent caries and anatomic form were the best of the studied factors with the least scores. Postoperative sensitivity was observed in cases with gingival recession caused due to periodontal complaints.^16^ Marginal adaptation was significantly different which could be attributed to small fracture of the prosthesis at the cavosurface margin junction. Another reason could be due to the cyclical forces occurring during mastication that can induce micromovement in the prosthesis.^15^ Surface texture of the prosthesis decreased overtime due to the loss of glaze and polish with usage. This may lead to accumulation of plaque and calculus and can lead to periodontal complications. Since these parameters showed statistically significant differences, the null hypothesis was rejected. Occlusion has a critical function in maintaining the simultaneous harmony between elements of the stomatognathic complex.^14^ Therefore, functional occlusal demands were also examined. There was also a lack of incisal guidance in a few cases which might be related to skeletal malocclusions. Mild skeletal malocclusions may be corrected dentally, however it is reliant on the tooth and material variables.^17^ There was lack of adequate cusp-fossa occlusion, resulting in participants being unable to chew finer objects correctly suggesting that micro-occlusion was affected. Such patients have a propensity to complain about functional insufficiency.^18^ Aesthetic issues were mostly connected to a midline mismatch or ceramic chipping. It was noticed that doctors concentrated more on matching the maxillary anterior gingival zeniths, however there were modest variations in the mandibular anterior gingival zeniths, leading to aesthetic problems in few individuals. With the improvements in technology assistance and dental materials sciences, there is a notion of minimally invasive dentistry. This focuses on approaches that conserve more tooth structure, decrease damage, and extend the life of restorations.^19^

Additionally, an obligatory reinforcement of appropriate oral hygiene habits such as bi-annual checkups is essential, that patients must adhere to for a successful and long lasting treatment outcome. Patient education regarding the treatment design, processes involved and results is indicated for greater patient compliance.^20^ This study presents several strengths that enhance the validity and relevance of its findings. Firstly, it is among the few retrospective clinical investigations specifically focused on the long-term outcomes of tooth-supported full mouth rehabilitations with the use of the standardized modified United States Public Health Service (mUSPHS) criteria. It allowed for objective and reproducible assessment of prosthesis performance. Additionally, the inclusion of both aesthetic and functional outcome measures offers a comprehensive evaluation beyond conventional structural metrics. The study's relatively large sample size and a consistent 3-year follow-up period strengthen the reliability of the results. Rigorous examiner calibration and high inter-rater reliability further reinforce the methodological robustness of the clinical evaluations. Despite its strengths, this study has certain limitations. The full mouth rehabilitations were performed by multiple clinicians with varying levels of technique and experience which could introduce variability in treatment planning, execution and outcomes. As a retrospective analysis, it depends on the accuracy and completeness of existing clinical records, which may introduce information bias. The absence of a control group limits the ability to draw causal inferences about the performance of specific materials or techniques used in full mouth rehabilitations. Although the modified USPHS criteria provides a standardized framework, it does not capture patient-related outcome measures such as comfort, masticatory efficiency, or aesthetic satisfaction. Additionally, the functional parameters such as occlusal discrepancies and incisal guidance were observational and not quantified using digital occlusal analyzers, which could have improved objectivity. Finally, being limited to a single institutional center may limit the generalizability of the findings to broader populations or different clinical settings.

## 4 Conclusion

This 3-year retrospective study highlights the overall clinical success of tooth-supported full mouth rehabilitations, with most parameters remaining stable over time. Significant changes in marginal adaptation and surface texture suggest functional wear effects. Common aesthetic and functional discrepancies emphasize the importance of precise planning and execution. Standardized evaluation and long-term follow-up are essential for optimizing rehabilitation outcomes.

## Patient consent

Written consent was obtained from each patient that participated in the study.

## Ethical clearance

Institutional ethical clearance IHEC/SDC/PROSTHO-2202/24/307.

## Sources of funding

Nil.

## Declaration of competing interest

The authors declare that they have no known competing financial interests or personal relationships that could have appeared to influence the work reported in this paper.
